# Algebraic Dynamic Programming over general data structures

**DOI:** 10.1186/1471-2105-16-S19-S2

**Published:** 2015-12-16

**Authors:** Christian Höner zu Siederdissen, Sonja J Prohaska, Peter F Stadler

**Affiliations:** 1 Bioinformatics Group, Department of Computer Science, Universität Leipzig, Härtelstraße 16-18, D-04107 Leipzig, Germany; 2 Department of Theoretical Chemistry, University of Vienna Währinger Straße 17, A-1090 Vienna, Austria; 3 Computational EvoDevo Group, Department of Computer Science, Universität Leipzig, Härtelstraße 16-18, D-04107 Leipzig, Germany; 4 Interdisciplinary Center for Bioinformatics, Universität Leipzig, Härtelstraße 16-18, D-04107 Leipzig, Germany; 5 Max Planck Institute for Mathematics in the Sciences, Inselstraße 22, D-04103 Leipzig, Germany; 6 Fraunhofer Institut for Cell Therapy and Immunology, Perlickstraße 1, D-04103 Leipzig, Germany; 7 Center for non-coding RNA in Technology and Health, Grønegårdsvej 3, DK-1870 Frederiksberg C, Denmark; 8 Santa Fe Institute, 1399 Hyde Park Rd., NM87501 Santa Fe, USA

**Keywords:** formal grammar, dynamic programming, gene duplications

## Abstract

**Background:**

Dynamic programming algorithms provide exact solutions to many problems in computational biology, such as sequence alignment, RNA folding, hidden Markov models (HMMs), and scoring of phylogenetic trees. Structurally analogous algorithms compute optimal solutions, evaluate score distributions, and perform stochastic sampling. This is explained in the theory of Algebraic Dynamic Programming (ADP) by a strict separation of state space traversal (usually represented by a context free grammar), scoring (encoded as an algebra), and choice rule. A key ingredient in this theory is the use of yield parsers that operate on the ordered input data structure, usually strings or ordered trees. The computation of ensemble properties, such as *a posteriori *probabilities of HMMs or partition functions in RNA folding, requires the combination of two distinct, but intimately related algorithms, known as the inside and the outside recursion. Only the inside recursions are covered by the classical ADP theory.

**Results:**

The ideas of ADP are generalized to a much wider scope of data structures by relaxing the concept of parsing. This allows us to formalize the conceptual complementarity of inside and outside variables in a natural way. We demonstrate that outside recursions are generically derivable from inside decomposition schemes. In addition to rephrasing the well-known algorithms for HMMs, pairwise sequence alignment, and RNA folding we show how the TSP and the shortest Hamiltonian path problem can be implemented efficiently in the extended ADP framework. As a showcase application we investigate the ancient evolution of HOX gene clusters in terms of shortest Hamiltonian paths.

**Conclusions:**

The generalized ADP framework presented here greatly facilitates the development and implementation of dynamic programming algorithms for a wide spectrum of applications.

## Background

Dynamic Programming (DP) over rich index sets provides solutions of a surprising number of combinatorial optimization problems. Even for NP-hard problems such as the Travelling Salesman Problem (TSP) exact solutions can be obtained for moderate size problems of practical interest. The corresponding algorithms, however, are usually specialized and use specific properties of the problem in an *ad hoc *manner that does not generalize particularly well.

Algebraic dynamic programming (ADP) [1] defines a high-level descriptive domain-specific language for dynamic programs over sequence data. The ADP framework allows extremely fast development even of quite complex algorithms by rigorously separating the traversal of the state space (by means of context free grammars, CFGs), scoring (in terms of suitable algebras), and selection of desired solutions. The use of CFGs to specify the state space is a particular strength of ADP since it allows the user to avoid indices and control structures altogether, thereby bypassing many of the pitfalls (and bugs) of usual implementations. Newer dialects of ADP [2,3] provide implementations with a running time performance close to what can be achieved by extensively hand-optimized versions, while still preserving most of the succinctness and high-level benefits of the original ADP language.

Sequence data is not the only type of data for which grammar-like dynamic programs are of interest. Inverse coupled rewrite systems (ICOREs) [4] allow the user to develop algorithms over both, sequence and tree-like data. While no implementation for these rewrite systems is available yet, they already simplify the initial development of algorithms. This is important in particular for tree-like data. Their non-sequential nature considerably complicates these algorithms. The grammar underlying the alignment of ncRNA family models with CMCompare [5], which simultaneously recurses over two trees, may serve as an example for the practical complications. There are compelling reasons to use DP approaches in particular when more information than just a single optimal solution is of interest. DP over sequences and trees readily allows the enumeration of all optimal solutions, and it offers generic ways to systematically investigate suboptimal solutions and to compute the probabilities of certain sub-solutions. Classified dynamic programming [6], furthermore, enables the simultaneous calculation of solutions with different class features via the evaluation algebra instead of constructing different grammars for each class.

An important research goal in the area of dynamic programming algorithms is the development of a framework that makes it easy to implement complex dynamic programs by combining small, simple, and reusable components. A first step in this direction was the introduction of grammar products [7], which greatly simplifies the specification of algorithms for sequence alignments and related dynamic programming tasks that take multiple strings as input. Several straightforward questions, however, still remain unanswered.

An important example is the relationship of Forward/Backward (in the context of linear grammars) [8] and Inside/Outside (in the context of CFGs) [9] algorithms. So far, the two variants need to be developed and implemented independently of each other. The close structural relationship of the two types of recursion has of course been noticed and used explicitly to facilitate algorithm design. The idea of "reverting" the inside production rules was used explicitly to explain backtracing and outside recursions in [10,11] for the RNA-RNA interaction problem and in [12] for RNA folding with pseudoknots, albeit without providing a general operational framework. In classical ADP the Inside algorithms are phrased as parsing an input string w.r.t. a given context free grammar. This is not possible in general for the Outside recursion because these operate, conceptually, on the complement of a substring. In some situations it is possible to rescue the ADP-style approach. For RNA folding, for example, Janssen [13] proposed to concatenate the suffix and the prefix in this order. The Outside recursion is then rephrased as a CFG on this modified string.

A second unsolved issue is that not all dynamic programming algorithms can be translated into the ADP framework in a straightforward manner. A classical example is the Travelling Salesman Problem (TSP). It is easily stated as follows: given a set *X *of cities and a matrix $d:X\times X\to {\mathbb{R}}_{+}$ of (not necessarily symmetric) distances between them, one looks for the tour (permutation) *π *on *X *that minimizes the tour length $f\left(\pi \right):=d_{\pi \left(n\right),\;\pi \left(1\right)}+\sum_{i=1}^{n-1}d_{\pi \left(i\right),\;\pi \left(i+1\right)}$. W.l.o.g., we may set *X *= {1, ..., *n*} and anchor the starting point of a tour at *π*(1) = 1. The well-known (exponential-time) DP solution for the TSP [14,15] operates on "sets with an interface" [*A, i*] representing the set of all tours starting in 1 ∈ *A*, then visiting all other cities in *A *exactly once and ending in *i ∈ A*. The length of the shortest path of this type is denoted by *f *([*A, i*]). For an optimal tour we have $f\left(\left[X,\;i\right]\right)+f\left(\left\langle j,\;i\right\rangle \right)\to \text{min}$, where $f\left(\left\langle i,\;1\right\rangle \right)=d_{1,\;i}$ is the length of the edge from *i *to 1. The values *f *([*A, i*]) satisfy the recursions

(1) $$f\left(\left[A,\;i\right]\right)=\underset{j\in A}{\text{min}}f\left(\left[A\backslash \left\{i\right\},\;j\right]\right)+f\left(\left\langle j,\;i\right\rangle \right)$$

since the shortest path through *A *to *i *must consist of a shortest path through *A *ending in some *j *∈ *A *and a final step from *j *to *i*.

A classical ADP formulation is impossible because the set *A *does not admit a string representation so that its subsets could be generated by a fixed set of productions. To split off a particular element {*i*} from *A*, for example, one requires a specific production rule of the form *A *→ (*A *\ {*i*}) ∪ {*i*}. This cannot be captured by a fixed CFG since the number of productions grows with the size of *A*.

Instead of relaxing the constraints on the number of productions we argue here that the solution to this conundrum can be resolved by a redefinition of the concept of parsing so that we can meaningfully write *A *→ *Ax *for the decomposition of a (nonempty) set into a subset with cardinality one less and the excluded single element. This restores one of the main advantages of ADP, namely the possibility to describe the state space traversal without explicit representation of indices. At the same time we will see below that the same formalism also yields a completely mechanical way to construct Outside recursions from the Inside algorithm. To this end we first consider the conceptually simple case of 1-dimensional and 2-dimensional linear grammars on strings using HMMs and pairwise sequence alignments as example. We then proceed to RNA folding as an example of a non-trivial CFG. The final step is to introduce an ADP-style formalism for non-trivial set-like data structures. Up to this point we keep our discussion informal and ignore several technical details. In section Theory we will then follow up with a much more abstract and precise account. Finally, we consider the probabilistic version of the shortest Hamiltonian path problem in the context of the early evolution of HOX gene clusters as a real-life application of our framework.

## Case studies

### HMMs and the Forward/Backward algorithms

A simple Hidden Markov Model (HMM) for detecting CpG islands in genomic DNA can specified as follows: (1) Each nucleotide position is contained either in a CpG island (state "+") or not (state "*−*"). (2) The probability that a nucleotide *p *follows *q *is given as $a_{pq}^{\sigma }$ and differs between the two states σ ∈ {+, -}. Furthermore we require a probability to switch from + to - of *q*^± ^and $q^{\mp }$, respectively. This yields transition probabilities $t_{i,i-1}^{++}=\left(1-q^{\pm }\right)a_{x_{i},x_{i-1}}^{+}$ and $t_{i,i-1}^{--}=\left(1-q^{\mp }\right)a_{x_{i},x_{i-1}}^{-}$ for the cases where the state remains unchanged + or - and $t_{i,i-1}^{+-}=q^{\mp }a_{x_{i},x_{i-1}}^{-}$ and $t_{i,i-1}^{-+}=q^{\pm }a_{x_{i},x_{i-1}}^{+}$ for the two possible state changes. Note that this formulation is much simpler than the usual HMM formalism since the emission probabilities are trivial here.

(2) $$\begin{array}{c} f^{+}\left[i\right]=f^{+}\left[i-1\right]t_{i,i-1}^{++}+f^{-}\left[i-1\right]t_{i,i-1}^{+-}\\ f^{-}\left[i\right]=f^{-}\left[i-1\right]t_{i,i-1}^{--}+f^{+}\left[i-1\right]t_{i,i-1}^{-+} \end{array}$$

The corresponding backward probabilities are

(3) $$\begin{array}{c} b^{+}\left[i\right]=b^{+}\left[i+1\right]t_{i,i+1}^{++}+b^{-}\left[i+1\right]t_{i,i+1}^{+-}\\ b^{-}\left[i\right]=b^{-}\left[i+1\right]t_{i,i+1}^{--}+b^{+}\left[i+1\right]t_{i,i+1}^{-+} \end{array}$$

This allows to compute $\mathbb{P}\left(i\in +\right)=f_{+}\left[i\right]b_{+}\left[i\right]$ and $\mathbb{P}\left(i\in -\right)=f_{-}\left[i\right]b_{-}\left[i\right]$.

In an ADP-style framework the forward recursion corresponds to the grammar with the productions

(4) $$\begin{array}{ccccccc} S & \to & P & | & M & & \\ P & \to & Pc & | & Mc & | & \varepsilon \\ M & \to & Pc & | & Mc & | & \varepsilon \end{array}$$

Apart from the formal start symbol *S*, it describes the two states as *P *and *M *and the possible transitions. The latter are both associated with prefixes [1..*i*] of the input strings up to some position *i*. The non-terminal *P *signifies that *i *has state +, while non-terminal *M *corresponds to the - state. The scoring of the productions *P *→ *Pc, P *→ *Mc*, etc., is relegated to a scoring algebra that encodes the multiplicativity of probabilities. Translated to recursion form, with indices 0 <*i *≤ *n *referring to positions in the input string and n denoting the length of the input, the forward recursions take on their usual form, see e.g. [[16], p. 51ff]:

(5) $$\begin{array}{lll} S_{n} & = & P_{n}+M_{n}\\ P_{i} & = & P_{i-1}\times c_{i}^{P\to Pc}+M_{i-1}\times c_{i}^{P\to Mc}+0\\ M_{i} & = & P_{i-1}\times c_{i}^{M\to Pc}+M_{i-1}\times c_{i}^{M\to Mc}+0\\ P_{0} & = & 1\;M_{0}=1 \end{array}$$

where $c_{i}^{P\to P_{c}}:=\left(1-q^{\pm }\right)a_{x_{i},x_{i-1}}^{+}$, etc., are the tabulated parameters of the HMM. The initialization *P*_0 _= *M*_0 _= 1 follows as the conditional probability of ending in the ϵ state after having read all input. Note that the strucuture of the recursion (5) is completely determined by the productions in equ.(4).

The backward recursion corresponds to a traversal of the input by means of suffixes. To each forward prefix [1..*i*] we have a matching sufix [*i*..*n*], where *n *is the length of the input. This overlap of corresponding prefix and sufix is just one indication that we might want to modify how we interpret the grammar (4). The fact that the scoring function explicitly refers to transitions, i.e., pairs of consecutive positions gives another hint. In this alternative picture we think of *P *and *M *as prefixes in which the last position takes on the role of a boundary ∂*M *and ∂*P*. Now we can think of the corresponding sufixes as the complements w.r.t. the input, i.e., to "forward objects" *P *and *M *we associate "backward objects" P^* ^and M^* ^so that, in terms of the index sets to which they refer, we have $P\cup P^{*}=S=\left\{1\ldots n\right\},M\cup M^{*}=S,\;P\cap P^{*}=\partial P$, and $M\cap M^{*}=\partial M$. Correspondingly, the terminals can be thought of as pairs of consecutive positions. This provides us with a mechanical way of scoring *c *as $c_{i}^{\pm }$ in the forward recursion and as $c_{i+1}^{\pm }$ in the backward recursion, since the terminal *c *defined for positions (*i *- 1; *i*) overlaps with the boundary of the forward objects *P *and *M *at *i *- 1, while the one defined on (*i*, *i *+ 1) overlaps on *i *+ 1 with the boundary of the backward objects P^* ^and M*.

Now, the corresponding backward grammar is, and should be compared closely to its progenitor (equ. 4):

(6) $$\begin{array}{ccccccc} \varepsilon^{*} & \to & P^{*} & | & M^{*} & & \\ P^{*} & \to & P^{*}c & | & M^{*}c & | & S^{*}\\ M^{*} & \to & P^{*}c & | & M^{*}c & | & S^{*} \end{array}$$

At first glance this notation looks awkward. One might have expected something like $P^{*}\to cP^{*}$. However, we will see below that for general CFGs the backward or outside objects P^* ^refer to the index set not covered by *P*. The notation P*c can be interpreted as the insertion of *c *at the right hand end of the "hole", i.e., as a left extension of the suffix. For completeness we translate eq.(6) into the corresponding recursions

(7) $$\begin{array}{c} \varepsilon_{0}^{*}=P_{0}^{*}+M_{0}^{*}\;(\text{outside final result})\\ P_{i}^{*}=P_{i+1}^{*}\times c_{i+1}^{P^{*}\to P^{*}c}+M_{i+1}^{*}\times c_{i+1}^{P^{*}\to M^{*}c}+0\\ M_{i}^{*}=P_{i+1}^{*}\times c_{i+1}^{M^{*}\to P^{*}c}+M_{i+1}^{*}\times c_{i+1}^{M^{*}\to M^{*}c}+0\\ P_{n}^{*}=1\;M_{n}^{*}=1 \end{array}$$

The *a posteriori *probability that sequence position *i *is in the + state is given by $P_{i}P_{i}^{*}$. In our frame-work, we obtain the complete list of these proabilities by using the probability scoring algebra and the formal production rule *S *→ P*P or, equivalently, *S *→ PP*. Writing *S *→ P*P as a production rule from the start symbol ties *P *and P^* ^together to be complementary, rather than independent non-terminals following the forward and backward grammar. Note that symbolically *S *→ P*P is no longer a linear production. It becomes useful, however, in our formalism to use the notation of production rules to specify any kind of decomposition of a data object. In this setting *S *→ P*P does makes sense: it defines the list of all complementary pairs of inside and outside objects, i.e., it serves as implicit specification of the outside object P^* ^to *P*. We will formally define this construct in the theory section.

### Prefix and sufix style sairwise sequence alignments

An analogous construction pertains to multiple sequence alignment. The only difference there is that now we operate simultaneously on multiple input tapes. For simplicity of exposition we consider only the pairwise alignment problem. Let us start with the well-known Needleman-Wunsch (NW) algorithm [17]. Starting from an empty alignment, we can think of it as extending an alignment *A *by either a (mis)match, an insertion, or a deletion. In grammar form this can be written as

(8) $$S\to A\;A\to A\left(\begin{array}{c} u\\ v \end{array}\right)|A\left(\begin{array}{c} u\\ - \end{array}\right)|A\left(\begin{array}{c} -\\ v \end{array}\right)|\left(\begin{array}{c} \varepsilon \\ \varepsilon \end{array}\right)$$

In contrast to the HMM example above, it is convenient here to interpret the string pairs *A *with an empty boundary: if *A *refers to the pair [1..*i*, 1*..j*] then *A*^∗ ^refers to [*i *+ 1*..n, j *+ 1*..m*] where *n *and *m*, resp. are the lengths of the input strings. The formal outside derivation, in terms of suffixes, of the NW algorithm is:

(9) $$\begin{array}{c} {\left(\begin{array}{c} \varepsilon \\ \varepsilon \end{array}\right)}^{*}\to A^{*}\\ A^{*}\to A^{*}\left(\begin{array}{c} u\\ v \end{array}\right)|A^{*}\left(\begin{array}{c} u\\ - \end{array}\right)|A^{*}\left(\begin{array}{c} -\\ v \end{array}\right)|S^{*} \end{array}$$

As for the HMM we think of A^* ^as a representation of the hole that is left over by *A*, and we read $A^{*}\left(\begin{array}{c} u\\ - \end{array}\right)$ as "fill $\left(\begin{array}{c} u\\ - \end{array}\right)$ into the hole *A*^∗ ^at its r.h.s. end. Clearly *S *→ *AA*^∗ ^and *S *→ *A*^∗^*A *refer to the complete global alignments with all possible "splitting constraints".

The outside productions (9) *look like *the suffix version of the NW algorithm. Writing the decomposition with full index information, however, shows that there is a suble difference: $A_{ij}\mapsto A_{i-1\;j-1}\left(\begin{array}{c} i\\ j \end{array}\right)$ transforms to $A_{ij}^{*}\mapsto A_{i+1,j+1}^{*}\left(\begin{array}{c} i+1\\ j+1 \end{array}\right)$, etc. This highlights the interpretation that A^* ^refers to the "hole" extending to the right from the positions *after i *and *j*, i.e., not including *i *and *j *itself. While this make little formal difference for the NW algorithm, it does have an important impact in the more complex case of Gotoh's algorithm for affine gap costs [18].

The different scoring of gap "opening" and "extension" implies that the gap-status at the end of a partial alignment must be known. To this end the CFG uses three non-terminals *M*, *D*, and *I *depending on whether the r.h.s. end of the alignment is a match state, a gap in the second sequence, or a gap in the first sequence. We ignore the issues of start (*S*) and stop $\left(\left(\begin{array}{c} \varepsilon \\ \varepsilon \end{array}\right)\right)$ symbols for the moment and return to them in the theory section below in a more systematic manner. The productions of the "body" of the recursions are of the form

(10) $$\begin{array}{ccccccccc} S & \to & M & | & D & | & I & & \\ M & \to & M\left(\begin{array}{l} u\\ v \end{array}\right) & | & D\left(\begin{array}{c} u\\ v \end{array}\right) & | & I\left(\begin{array}{c} u\\ v \end{array}\right) & | & \left(\begin{array}{c} \varepsilon \\ \varepsilon \end{array}\right)\\ D & \to & M\left(\begin{array}{c} u\\ - \end{array}\right) & | & D\left(\begin{array}{c} u\\ \cdot \end{array}\right) & | & I\left(\begin{array}{c} u\\ - \end{array}\right) & | & \left(\begin{array}{c} \varepsilon \\ \varepsilon \end{array}\right)\\ I & \to & M\left(\begin{array}{c} -\\ v \end{array}\right) & | & |D\left(\begin{array}{c} -\\ v \end{array}\right) & | & I\left(\begin{array}{c} \cdot \\ v \end{array}\right) & | & \left(\begin{array}{c} \varepsilon \\ \varepsilon \end{array}\right) \end{array}$$

where *u *and *v *denote terminal symbols. '*−*' corresponds to gap opening, while '.' denotes the (differently scored) gap extension.

The interpretation of the non-terminals *M*, *D*, and *I *is determined by the last column of the prefix alignment: it ends in a (mis)match, a deletion, or an insertion, respectively. In contrast to the HMM example of the previous section we do not score transitions here. Thus we interpret the non-terminals as boundary-free. Hence M^* ^becomes a suffix object complementing a prefix alignment that ends in a (mis)match. Note that this does *not *mean that *M*^∗ ^itself ends in a (mis)match. Because *M *, and thus *M*^∗ ^are boundary-free, the corresponding alignments do not overlap. As a consequence, their scores can be added. This property is required for the evaluation algebras to behave properly.

Transforming the linear grammar eq.(10) into its outside recursions yields

(11) $$\begin{array}{ccccccccc} {\left(\begin{array}{c} \varepsilon \\ \varepsilon \end{array}\right)}^{*} & \to & M^{*} & | & D^{*} & | & I^{*} & & \\ M^{*} & \to & M^{*}\left(\begin{array}{c} u\\ v \end{array}\right) & | & D^{*}\left(\begin{array}{c} u\\ - \end{array}\right) & | & I^{*}\left(\begin{array}{c} -\\ v \end{array}\right) & | & S^{*}\\ D^{*} & \to & M^{*}\left(\begin{array}{c} u\\ v \end{array}\right) & | & D^{*}\left(\begin{array}{c} u\\ \cdot \end{array}\right) & | & I^{*}\left(\begin{array}{c} -\\ v \end{array}\right) & | & S^{*}\\ I^{*} & \to & M^{*}\left(\begin{array}{c} u\\ v \end{array}\right) & | & D^{*}\left(\begin{array}{c} u\\ - \end{array}\right) & | & I^{*}\left(\begin{array}{c} .\\ v \end{array}\right) & | & S^{*} \end{array}$$

A first glance, this grammar looks odd. It is not the grammar for the suffix-version of Gotoh's algorithm. Instead, it refers to a rather unusual way of solving the affine gap cost problem. Here the distinction is not made between opening or extending a gap, but rather between closing or extending it. The nonterminals on the r.h.s. of the rule thus refer to the type of alignment that is reached *after *extending the one on the l.h.s. of the rule by the terminal symbol appearing on the r.h.s. Since our forward recursion (10) is set up to separately score gap opening, i.e., the left-most gapped position in the alignment, the same must be true for the backward recursion. Since it proceeds from right to left on the input string, we naturally arrive at the algorithmic variant that scores gap closing separately. The corresponding non-terminals therefore depend on how the alignment is continued in the subsequent step.

On a more technical note, the conversion of (10) to (11) does *not *break the signature type isomorphism between inside and outside variant. Where previously the rule $D\to I\left(\begin{array}{c} u\\ - \end{array}\right)$ makes use of an attribute function with type $\Gamma \times \left(\begin{array}{c} Char\\ - \end{array}\right)\to \Gamma$ for evaluation, this type now corresponds to the rule $I^{*}\to D^{*}\left(\begin{array}{c} u\\ - \end{array}\right)$. Here Γ is the type of the evaluated parse, e.g. a probability for SCFGs or an energy or a partition function for RNA folding.

One obtains the probability of matching each pair $\left(\begin{array}{c} i\\ j \end{array}\right)$ via *S *→ *MM*^∗ ^*≡ M*^∗^*M*. Each *M_ij _*indicates an alignment ending in a match with indices $\left(\begin{array}{c} i\\ j \end{array}\right)$, while $M_{ij}^{*}$ yields alignments where the matching characters at $\left(\begin{array}{c} i\\ j \end{array}\right)$ "have just been transitioned from".

### Inside and outside: RNA folding

State-of-the-art RNA folding programs (as implemented e.g. in the ViennaRNA package [19]) incorporate the nearest-neighbour model. For the purpose of a more compact presentation, we restrict ourselves here to the much simpler model(12)

The full model [20] amounts to a more complicated decomposition of secondary structure enclosed by a base pair (2nd decomposition). In a more conventional, but mnemonically less pleasing form the productions in equ.(12) read

(13) $$\begin{array}{ccc} S\to U & \;U\to cU|BU|\varepsilon & B\to cUc\prime \end{array}$$

This is a conventional CFG acting on the input string, here an RNA sequence. As usual we write *c*...*c' *to mean all 6 combinations of canonical base pairs gc, cg, au, ua, gu, and ug. In terms of recursions with explicit indices, its interpretation is

(14) $$\begin{array}{cccc} S_{1,\;N} & \mapsto & U_{1,\;N} & \\ U_{ij} & \mapsto & \varepsilon_{i>j} & \text{empty parse}\\ U_{ij} & \mapsto & c_{i}U_{i+1,j} & U_{ij}\mapsto B_{ik}U_{k+1,j}\\ B_{ij} & \mapsto & c_{i}U_{i+1,j-1}c_{j}^{\prime } & \end{array}$$

The indices here explicitly designate a substring of the input to which a particular non-terminal or terminal symbol refers.

The non-terminal *S*, which refers to unconstrained secondary structures, has an empty boundary. In contrast, it is natural to think about *B_ij _*as having the closing base pair ⟨*i*, *j*⟩ as its boundary. The reason is that the standard energy model for RNAs in general evaluates the "loop" enclosed by ⟨*i*, *j*⟩ rather than the pair itself. This is also true for corresponding outside objects, i.e., *(i, j) *naturally contributes both inside and outside "loops" that it delimits. In the natural way to define outside objects this is different for *S *and *B*. The complement of *S_ij _*is $S_{i-1,j+1}^{*}$ and refers to secondary structures on [1..*i *- 1] ∪ [*j *+ 1*..n*]. In contrast, the complement of *B_ij _*is $B_{ij}^{*}$, referring to secondary structures on the union [1..*i*] ∪ [*j..n*]. Thus *S *∩ *S*^∗ ^= *∅*, while B ∩ B^* ^is the common enclosing base pair.

**Figure 1:**
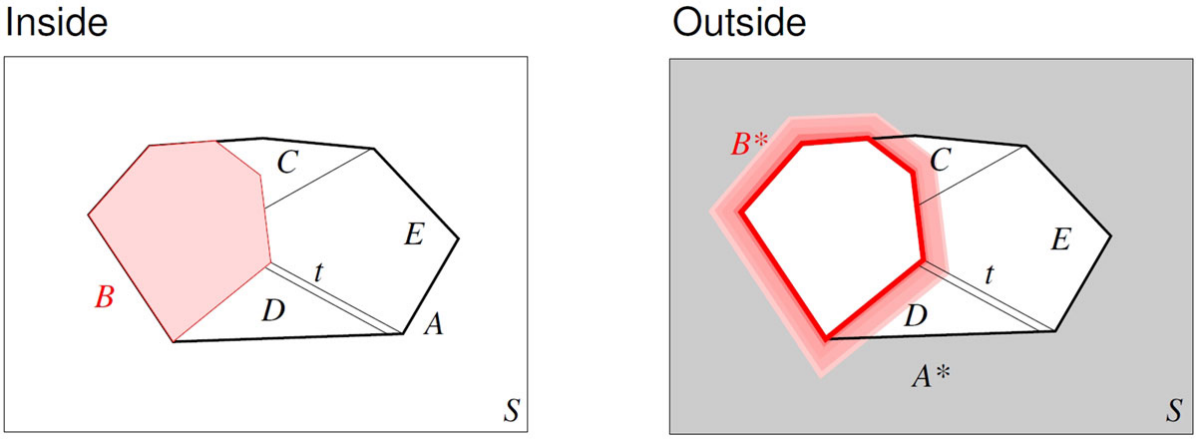
**Conversion of Inside productions into decompositions of the corresponding Outside objects**. A production rule *A *→ ... describes in which ways *A *can be partitioned, here into 5 parts *B*, *C*, *D*, *E*, and *t*. The corresponding outside objects *A*^∗ ^and *B*^∗ ^are the complements of *A *and *B *w.r.t. to input, represented by the box *S*. A rule *B*^∗ ^→ ... describes the decomposition of *B*^∗^, i.e., *S *\ *B*. Borrowing from *A *→ *BCDEt *we see that *B*^∗ ^consists of complement *A*^∗ ^of *A *as well as the pars of *A *with the exception of *B *itself.

In order to understand how the inside grammar (14) gives rise to recursions for the outside variables S^* ^and B^* ^we first consider the conceptual picture illustrated in Figure 1. Since a production corresponds to a decomposition of an object in smaller constituents, we may pick one of these parts and ask for a decomposition of its complement. The complement of course is formed with respect to some "ground set", in our case the complete input.

This idea is easily made precise for an arbitrary CFG. Consider a derivation of the form *A → αBβ*, where *B *is a non-terminal and *α*, *β *are strings of terminals and non-terminals. Here *A *is decomposed into *B *as well as the rest *α *∪ *β*. Looking at the complements, therefore, B^* ^consists of A^* ^and the rest *α *∪ *β*. Since *A *and *B *are intervals in a CFG setting, their complements are disjoint unions of a prefix and a suffix of the input. Thus *A → αBβ *transforms to

(15) $$A\to \alpha B\beta ⇝B^{*}\to \alpha A^{*}\beta$$

where the string of forward (non)terminals *α *is filled from the left into the hole of A^* ^and *β *is filled in from the right. This is always well-defined because the outside rules always contain exactly one outside non-terminal on both the l.h.s. and the r.h.s. of the derived rule. For an inside production *A → γ *we obtain one outside production for every non-terminal *B ∈ γ*. Thus we have to split *γ *= *αBβ *for all non-terminals *B ∈ γ*. This can easily be achieved in a completely mechanical way. For the RNA example this yields

(16) $$\begin{array}{c} \varepsilon^{*}\to U^{*}\\ U^{*}\to cU^{*}|BU^{*}|cB^{*}c\prime |S^{*}\\ B^{*}\to U^{*}U \end{array}$$

In diagrammatic form this can be written in the following way(17)

It is instructive to translate this outside grammar-style into a more conventional recursive form that explicitly exposes the indices:

(18) $$\begin{array}{c} U_{ij}^{*}\mapsto c_{i-1}U_{i-1,j}^{*}\\ U_{ij}^{*}\mapsto c_{i-1}B_{i-1,j+1}^{*}c_{j+1}^{\prime }\\ U_{ij}^{*}\mapsto B_{k,i-1}U_{k,j}^{*}\;\;k<i\\ B_{ij}^{*}\mapsto U_{i,k}^{*}U_{j+1,k}\;\;k>j\\ U_{ij}^{*}\mapsto \in_{i=1,j=n} \end{array}$$

We can now derive McCaskill's algorithm [21] for computing the base pairing probabilities by (1) writing down the inside grammar (see e.g. [22] for several variants), (2) specifying the evaluation algebra for the partition function (see theory section), (3) generating the outside recursions, and (4) producing the list *S *→ *BB*^∗^.

### Shortest Hamiltonian paths

We now leave the realm of classical ADP behind and consider dynamic programming algorithms on unordered data structures. This most clearly requires us to rethink what we mean by a "grammar" and by "parsing". Since shortest Hamiltonian paths play a key role in the show-case example later on we introduce them here as an illustrative example.

SHORTEST HAMILTONIAN PATH (SHP). Given a graph *G *with vertex set *V *and edge set *E *and a dissimilarity matrix $d:E\to {\mathbb{R}}_{+}$ the task is to find a path *π *in *V *that runs through every vertex and minimizes the total length $\ell \left(\pi \right)=\sum_{i=1}^{n-1}d_{\pi \left(i\right)\pi \left(i+1\right)}.$.

SHP is a well known NP-complete combinatorial optimization problem. It can be solved exactly by a simple DP algorithm [14,15], which of course in general has exponential runtime. Denote by [*i, A, j*] with *i, j *∈ *A *⊆ *V *the set of all Hamiltonian paths through a subset *A *that have *i *and *j *as its endpoints. For every *k *∈ *A *\ {*i, j*} we can decompose [*i, A, j*] into the edge ⟨*k*, *j*⟩ and the set [*i, A, k*] of shorter Hamiltonian paths. We can write this decomposition in the form

(19) A→Av

in complete analogy to a linear grammar. The point of this section is that we can make this analogy precise and useful.

Let us first consider this rule for an arbitrary set. Then *A → Av *tells us to split off a single element (atom) from *A*. On string data structures there is essentially only a single way of doing this, namely to remove a single-character suffix. Removal of a prefix would be encoded by *A → vA*, i.e. a distinct production. On sets, we now have |*A*| possibilities, i.e., we obtain a list of possible decompositions. Since the underlying data structure has no intrinsic order, the productions *A → Av *and *A → vA *are of course equivalent. This is not different from CFGs, in fact: A production of the form *A → BC *returns |*A*| + 1 partitionings of *A *into a prefix *B *and a suffix *C*. Of course *A → BC *also makes perfect sense for sets: *B *and *C *now form the bipartitions of *A*, i.e., there are 2*^|A| ^*alternative decompositions. The only difference between strings and sets thus is the number of alternative parses.

In the SHP example there is a further complication: we have to keep track of the end points *i *and *j*. Instead of regular sets, we thus have an additional "punctuation" structure that defines a start and end point. The parsing rule *A → Av *now has to know that (i) the end point has to be split off, and in doing so, (ii) a new end-point distinct from the startpoint has to be determined so that we obtain again a properly punctuated set. Furthermore it becomes the parser's job to know that (iii) the terminal *v *is the connection between new and old endpoint, rather than just a split-off vertex. We note in passing that simply re-interpreting the punctuated sets *A *as connected components so that neither end point is a cut vertex of the input graph may increase practical efficiency since it prunes early those subsets through which no path connecting the end points can be constructed. Whether we want to think of the startand endpoints as distinct features, or whether either one can be used to decompose the set, is a matter of modelling and defines two distinct data types.

Formally, furthermore, we see that there is a second type of decomposition operations that seems useful in general ADP. We may simply write

(20) V→A

and assume that our machinery knows that *V *is an unstructured set, while *A *is a set with start and endpoint. The trivial-looking rule therefore provides a list of |*V*|(|*V*| − 1) or |*V*|(|*V*| − 1)/2 punctuated sets, depending on whether start and end are distinguished or not.

Of course we can immediately construct *A*^∗ ^as a complementary punctuated set with the same endpoint, i.e., so that [*i, A, j*][*j, A*^∗^, *k*] overlap in the common point *j*. We then have

(21) $$A^{*}\to A^{*}v$$

as the corresponding grammar for the outside objects.

For the Hamiltonian paths through a particular adjacency (terminal) *v *we can now write *V → AvA*^∗^, i.e., this is a top-level decomposition of the start symbol, i.e., acting on the input string. We simply have to use as scoring algebra the multiplication of partition functions from *A *and *A*^∗ ^(as *S *\ *A*) and fix the Boltzmann-weights $Z\left(\left\langle i,j\right\rangle \right)=\text{exp}\left(-\alpha d_{ij}\right)$ for the terminals. This computes the *a posteriori *probability of observing an adjacency *i *~ *j *in the path with fixed endpoints *p *and *q*, i.e.,

(22) $$\begin{array}{c} P\left(i\sim j|p,q\right)=\frac{1}{Z\left(S_{pq}\right)}\\ \underset{A\subset S}{\sum }Z\left(\left[p,\;A,\;i\right]\right)Z\left(\left\langle i,\;j\right\rangle \right)Z\left(\left[j,\;S\backslash A,\;q\right]\right). \end{array}$$

in the index-based notation. Similarly, *P *(ends=*p, q*) = *Z*(*S_pq_*)*/Z*(*S*) provides us with the probabilities that the path ends in *p *and *q *and $P\left(\text{end}=\text{p}\right)=\sum_{q}P\left(\text{ends}=p,q\right)$ measures how frequently we expect *p *to be an end point of a path. We make use here of the distinction between the start symbol *S*, which refers to a set without boundary, and *S_pq_*, a set [*p, A, q*] with two boundary points *p *and *q *defining the end points of the paths running through it. Thus *S → S_pq _*with "sum" as choice function and the identity attribute function (attribute functions of an algebra evaluate individual parses) yields *Z*(*S*), summing over all (*p, q*). On the other hand, *S → S_pq _*with the identity choice function and attribute function $\lambda z.\frac{z}{Z\left(S\right)}$ (where λz.B denotes an anonymous function with body B  expecting *z *as its single argument) returns a list of all (*p, q*) start/end points, together with the probabilities that these points are start and end point. To obtain the end probabilities in our framework we need an additional type of non-terminals, say *S_p_*, that have only a single point as boundary, i.e., it refers to sets of the form [*p, A*]. The production *S → S_p _*with the identity choice and attribute $\lambda z.\frac{z}{Z\left(S\right)}$ then yields the desired end probabilities. *S_p _→ S_pq _*folds over all *S_ip _*and *S_pi _*for all *i*, as we now do not distinguish between a 'starting' and 'ending' point in a path for *S_p_*.

## The formal framework

The key ingredient in our approach is to generalize grammars to decomposition schemes of a wide range of data models by redefining what exactly parsers do. Let us start with making explicit how this works in classical ADP, i.e., for (context free) grammars on strings:

1 Each (non)terminal corresponds to a substring.

2 Each terminal symbol matches a single character of the input string.

3 Each production defines a (list of) partitions. More precisely, the substrings corresponding to the r.h.s. of the production partition the substring corresponding to the single non-terminal on its l.h.s.

4 The partition is order preserving, i.e., the sequence of symbols on the r.h.s. matches the order of the corresponding substrings on the input string.

We have seen that it may be convenient already in the realm of strings to give up some of these requirements e.g. to treat problems in which terminals are naturally interpreted as transitions between adjacent positions as in the HMM case.

To formalize this we introduce objects with boundaries and allow that objects on the r.h.s. of a product overlap in their boundaries in a certain way. To this end we define for each object *A *its boundary *∂A *and its interior int(*A*) := *A *\ *∂A*. An object together with its boundary is denoted by [*A, ∂A*]. We also allow terminals to match more than an atomic constituent of the input data structure. An example are the pairs of adjacent characters in the HMM case and the edges of input graphs in the Hamiltonian paths. The productions take on the form of decomposition rules

(23) $$\left[A,\;\partial A\right]\to \underset{i}{⋃}\left[A_{i},\;\partial A_{i}\right]$$

for which we require the following properties:

(C1) ${⋃}_{i}A_{i}=A$, i.e., the decomposition products of *A *form a covering of *A*.

(C2) int *A_i _*∩ int *A_j _*≠ ∅ implies *i *= *j*, i.e., the interiors of the parts are disjoint.

(C3) int *A_i _*⊆ int *A*, i.e., the interiors behave like isotonic functions.

Note that these axioms recover the partition-style parsing if all data objects have empty boundaries. In this case (C3) follows from (C2). Whether we treat the ${⋃}_{i}\left[A_{i},\;\partial A_{i}\right]$ as an ordered list or as a multiset (or as something inbetween) depends on the intrinsic internal order structure of *A*. Here we have encountered only total orders (on strings) and anti-chains (for sets). Non-trivial partial orders however may become important when dealing with tree structures.

The parsers can infer much of the necessary index handling from considering meta-rules for handling adjacent boundaries. For instance, it will be useful in many cases to declare that boundaries of adjacent objects can overlap only if they coincide. In the RNA example enclosing base pairs appear as object boundaries. Semantically, it make no sense to allow overlap of base pairs at one end but not at the other. In other cases, however, it is useful to require only that *∂A_i _*⊆ *A_j _*or *vice versa*. This is the case in the HMM and SHP example, where we might want to interpret the terminals as transitions and edges as having empty interior and thus consisting of boundary only. DP algorithms where the index arithmetic in the decompositions is even more complex for instance appear in RNAwolf [23] and in the context of the coloring problems associated with RNA design in [24].

As we use complementarity w.r.t. the input to define the outside objects we have

(24) $$S\to \left[A,\;\partial A\right]\left[A^{*},\;\partial A^{*}\right].$$

since the start symbol *S *refers to the unprocessed, complete input. By construction, therefore, *∂A *= *∂A*^∗^, and *A *and *A*^∗ ^overlap at the boundary. The same complementarity is the basis for deriving the outside recursion in a well-defined manner from the inside recursion using equ.(15) in the ordered case or even simpler

(25) $$A\to \alpha B\gamma ⇝B^{*}\to \alpha A^{*}\gamma$$

where *γ *is a set of terminals and non-terminals. By construction there is exactly one syntactic outside variable on the r.h.s. of an outside production rule. All other symbols on the r.h.s. are either terminal symbols or inside symbols. From the perspective of the outside variables, they behave as "syntactic terminals", i.e., in a combined inside/outside grammar none of their derivations ever reaches an outside variable. As an immediate consequence we conclude that the outside grammar is bi-linear (and even linear in the unordered case) in its outside syntactic variables. Given a description of parsing we can now use the conventional ADP framework.

### Start symbols, stop symbols, and normalized grammars

The start symbol *S *and stop symbol *ε *are complementary in a natural manner: *S *refers to the complete, unprocessed input, while *ε *recognizes that the input has been used up and there is nothing left to parse. Thus *S *≅ *ε*^∗ ^and *ε *≅ *S*^∗^. More precisely, each inside rule of the form *S → A *has a corresponding outside rule *A*^∗ ^→ *σ*^∗^, and each inside rule *A → ε *yields translates into the outside rule *E*^∗ ^→ *A*^∗^. We say that a grammar is *normalized *if

(1) Every production rule *S → α *with the start symbol *S *on the l.h.s. has a single non-terminal (or syntactic variable) on the r.h.s.

(2) All production rules with only terminals on the r.h.s. have just a single *ε *(and no other symbol) on the r.h.s.

While it is not strictly necessary to work with normalized grammars, they are practically convenient because normalization guarantees that start and stop rules that isomorphic evaluation function types in their respective inside and outside version. It is easy to see that if G  is normalized, then its outside variant $G^{*}$ is also normalized.

As an illustration we complete here the outside recursions for Gotoh's alignment algorithm. Recalling eqns. (10) and (11) we first have to explain the rules for the outside start symbol. From the inside rules *M *→ *ε*, *D *→ *ε*, and *D *→ *ε *we obtain the expected productions *E*^∗ ^→ *M*^∗ ^| *D*^∗ ^|*I*^∗^. Furthermore, *S *→ *M *|*D*| *I *yields the termination rules *M*^∗ ^→ *σ*^∗^, *D*^∗ ^→ *σ*^∗^, and *I*^∗ ^→ *σ*^∗^. The final outside variant of Gotohs algorithm now reads:

(26) $$\begin{array}{ccccccccc} E^{*} & \to & M^{*} & | & D^{*} & | & I^{*} & & \\ M^{*} & \to & M^{*}\left(\begin{array}{c} u\\ v \end{array}\right) & | & D^{*}\left(\begin{array}{c} u\\ - \end{array}\right) & | & I^{*}\left(\begin{array}{c} -\\ v \end{array}\right) & | & {\left(\begin{array}{c} \sigma \\ \sigma \end{array}\right)}^{*}\\ D^{*} & \to & M^{*}\left(\begin{array}{c} u\\ v \end{array}\right) & | & D^{*}\left(\begin{array}{c} u\\ \cdot \end{array}\right) & | & I^{*}\left(\begin{array}{c} -\\ v \end{array}\right) & | & {\left(\begin{array}{c} \sigma \\ \sigma \end{array}\right)}^{*}\\ I^{*} & \to & M^{*}\left(\begin{array}{c} u\\ v \end{array}\right) & | & D^{*}\left(\begin{array}{c} u\\ - \end{array}\right) & | & I^{*}\left(\begin{array}{c} \cdot \\ v \end{array}\right) & | & {\left(\begin{array}{c} \sigma \\ \sigma \end{array}\right)}^{*} \end{array}$$

In the context of multi-dimensional grammars for alignments we have to deal with gap symbols referring to an empty input on one or more input tapes. Although gaps are superficially similar to stop symbols they only appear in the context of actually parsing an input symbol, albeit on another tape, they are handled just like any other character-parsing terminal. In particular they do not give rise to a start symbol in the outside grammar.

### Combining Inside and Outside variables: *a posteriori *probabilities

ADP grammars come with a signature that describes the types of the attribute functions attached to each production rule. One of the fringe benefits of constructing the outside grammar automatically according to equ.(15) or equ.(25) is that the inside and outside grammars are guaranteed to be isomorphic with respect to their signature. This, in turn, simplifies re-use of evaluation algebras between inside and outside.

The formal production *S → P*^∗^*P ≡ PP*^∗^, as an inside rule, states that parses *p*^∗ ^should be combined with corresponding parses *p*. This is, however not a normal context-free rule. If *P *and its outside complement *P*^∗ ^come from a linear grammar with a set-based index type, then the intuition is correct. For a linear grammar on a string index type, this looks intuitively correct, but the underlying index type does not admit a context-free grammar at all as linear grammars have a fixed left or right end point for each sub-parse. This issue can be resolved by observing that *S → PP*^∗ ^in an inside context translates into generating all possibilities of splitting (the index representation of) the complete input into an inside and an outside part. For linear string grammars there are the *O*(*n*) ways to split 0 *≤ k ≤ n *at *k*; for string CFGs there are *O*(*n*^2^) ways to split 0 *≤ k ≤ l ≤ n *at (*k, l*), and linear set grammars yield *O*(2*^n^*) different split points of a set with *n *bit. For multi-tape grammars, the behaviour follows in an analog fashion.

The production *S → PP*^∗ ^requires an attribute function evaluating each parse (*p, p*^∗^) ∈ *P, P*^∗^, and a choice function. The evaluation algebra for probabilities or partition functions (which are essentially unnormalized probabilities) comprises multiplication for terms appearing in decompositions and addition for alternative productions from the same non-terminal. For the terminals, score values are tabulated as parameters. In the case of RNA folding, these are the Boltzmann factors exp(*−E*(*t*)*/RT *) of the energies *E*(*t*) associated with the terminal *t*. For the RNA toy model of Sec. we have *E*(*t*) = 1 for a base pair terminal and *E*(*t*) = 0 for an unpaired terminal. In the more realistic setting, the loop energies of the Turner model are used. The practical evaluation will typically be along the lines of $\lambda pq.\frac{p\times q}{Z}$ to yield the probability, where the normalization constant *Z *is obtained by evaluating the start nonterminal.

## Application: shortest Hamiltonian paths and gene cluster histories

Local duplication of DNA segments via unequal crossover is the most plausible mechanism for the emergence and expansion of local clusters of evolutionary related genes. Although there are polynomialtime algorithms to reconstruct duplication trees from pairwise evolutionary distance data [25] this approach often fails to resolve the ancient history of gene clusters. The reason is the limited amount of phylogenetic information in a single gene. The situation is often aggravated by the extreme time scales leading to a decay of the phylogenetic signal so that only a few, very well-conserved sequence domains can be compared. A large number of trees then fits the data almost equally well. A meaningful analysis of the phylogeny thus must resort to some form of summary information that is less detailed than a fully resolved duplication tree. In the absence of genome rearrangements, and if duplication events are restricted to copying single genes to adjacent positions, we expect genetic distance to vary monotonically with genomic distance, i.e., we expect - at least approximately - to have *d_ik _≥ *max(*d_ij_, d_jk_*) whenever gene *j *lies between *i *and *k *on the genome. The same is true if gene duplications arise by unequal crossover and subsequent divergence rates are comparable. This so-called Robinson property ensures that a shortest Hamiltonian path through the genetic distance matrix conforms to the linear arrangement of the genes on the genome [26]. A mathematically more precise exposition of the role of short Hamiltonian paths in clusters of paralogous genes can be found in a forthcoming manuscript [27].

The same high noise level that suggests to avoid duplication trees should also make us distrust the shortest path. More robust results can be expected by considering the information on the ensemble of all Hamiltonian paths. We therefore compute the probabilities *P *(*i *~ *j*) of the individual adjacencies assuming a Boltzmann weighting *p*(*π*) ∝ exp(−*ℓ*(*π*)*/RT *) of the Hamiltonian paths *π*. The parameter *T *is a fictitious temperature governing the relative importance of short *versus *long paths *π*. For *T → *0 we focus on the (co)optimal paths only, while *T → ∞ *leads to a uniform distribution of adjacencies. The normalization constant is conveniently set to $R=\left(n-1\right)\bar{d}$, where $\bar{d}$ is the average of the genetic distance between genes. The path length ℓ(*π*) plays the role of the energy in the partition function of RNA secondary structures and of the dissimilarity score in probabilistic alignment algorithms. As we have seen above, the The ADP-style framework provides us with an easy and efficient way to compute the probabilites *P *(*i *~ *j*) of adjacencies along short Hamiltonian paths and the probabilities *P *(end = *i*) that gene *i *is the endpoint of a short path. In intact clusters we expect that the ends of genomic cluster also appear as the most probable ends of the Hamiltonian paths. High probabilities in the interior, by contrast, are a good indicator of rearrangments.

Hox genes are ancient regulators originating from a single Hox gene in the metazoan ancestor. Over the course of animal evolution the Hox cluster gradually expanded to 14 genes in the vertebrate ancestor [28]. Timing and positioning of Hox gene expression along the body axis of an embryo is co-linear with the genomic arrangement in most species. Only the 60 amino acids of the so-called homeodomain can be reliably compared at the extreme evolutionary distances involved in the evolution of the Hox system. We quantitatively measure the genetic distance of the homeodomain sequences either using the Hamming distance, i.e. the number of different amino-acids, or the transformation *dab *= *s*(*a, a*) + *s*(*b, b*) *− *2*s*(*a, b*) of the BLOSSUM45 similarity scores.

**Figure 2:**
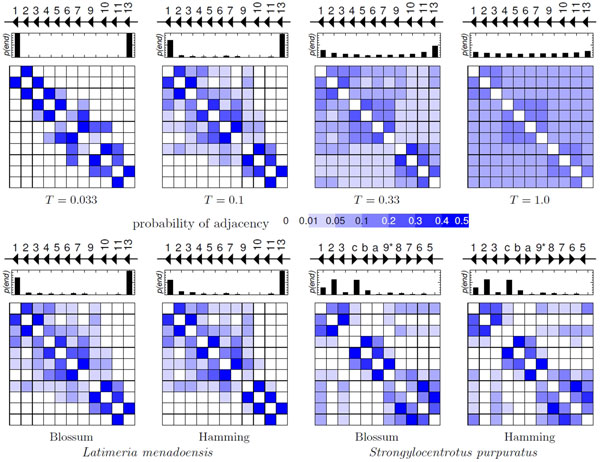
**Analysis of two Hox gene cluster in terms of shortest Hamiltonian paths w**.r.t. genetic distance. Small diagrams indicate the genomic order and reading direction of the genes (black triangles). The small histograms show the probability that a gene is endpoint of a Hamiltonian path, the square panels below display the posterior probabilities of adjacencies of Hox genes along shortest Hamiltonian paths w.r.t. to genetic distance. **Top: **effect of the temperature parameter *T *for distances between *Latimeria menadoensis *homeobox sequences. **Below: **Comparison of adjacencies for two different metrics (Hamming distance, and BLOSSUM45 derived dissimilarities) in *L. menadoensis *(left) and *S. purpuratus *for *T *= 0.1 to emphasize the structure of the ambiguities. Gene names are abbreviated 1-13 = HoxA1-HoxA13 for *L. menadoensis *and 1-8 = HoxA1-HoxA8, 9* = HoxA9/10, a-c = HoxA11/13a-HoxA11/13c for *S. purpuratus*.

We analyzed here the Hox A cluster of *Latimeria menadoensis *(famous as a particularly slowly evolving "living fossil"), which has sufferered the fewest gene losses among vertebrates. The 11 HoxA genes are arranged in the same order and orientation reflecting the gene order of the vertebrate ancestor: HoxA13, HoxA11 to HoxA9 and HoxA7 to HoxA1. In contrast, the Hox cluster of the sea urchin *Strongylocentrus purpuratus *has undergone fairly recent rear-rangements of its gene order [29]. The putative ancestral cluster most likely had three anterior, five middle and one to five posterior genes. The exact number is not known because the time point of the posterior expansion is uncertain. The gene set of *S. purpuratus *is reminiscent of the ancestral configuration. However, it reveals a gene order wherein the anterior genes (Hox1, Hox2 and Hox3) lie nearest to the posterior genes (Hox11/13c, Hox11/13b, Hox11/13a and Hox9/10), see Figure 2. Several rearrangement schemes have been proposed, a minimum of one translocation, two gene inversions and the loss of Hox4 is required to reach the current configuration. Figure 2 shows the posterior probabilities of adjacencies. Both, the coelacanth and the sea urchin examples reflect the well-known clustering into anterior (Hox1-3), middle group genes (Hox4-8), and posterior ones (Hox9-13). The shortest Hamiltonian paths in *L. menadoensis *connect the Hox genes in their genomic order. The high endpoint probability values *p*(hoxA1, *T *= 0.1) = 0.699 and *p*(hoxA13, *T *= 0.1) = 0.960 correctly identify HoxA1 and HoxA13 as cluster endpoints. In the sea urchin, however, we see adjacencies connecting the anterior subcluster (Hox1-3) with the genomic end of the cluster, i.e., the middle group genes (Hox8-Hox5). This is indicative of the recent cluster rearrangement. With a factor of about 2 the endpoint probability value favors hoxA2 over hoxA1 (the true endpoint). Note also that independent posterior expansion in Chordata (such as *L. menadoensis*) and Ambulacraria (such *S. purpuratus*) has lead to paralogs with greater genetic distance than observed among the anterior and middle group genes.

## Discussion

We have taken here the first step towards extending algebraic dynamic programming (ADP) beyond the realm of string-like data structures. Our focus is an efficient, yet notationally friendly way to treat DP on unordered sets. As a showcase application we used ADP on sets to demonstrate that statistics over Hamiltonian paths can be computed efficiently as means of analyzing the ancient evolution of gene clusters. This extension of ADP builds on the same foundation (namely ADPfusion [2]) as our grammar product formalism [7,30]. The key idea is to redefine the rules of parsing to match the natural subdivisions of the data type that now may be much more general than strings. In the case of sets, these are bipartitions and the splitting of individual elements, rather than the subdivision of an interval or the removal of a boundary element that are at the heart of string grammars. A particularly useful feature of our work is the ADP-style implementation and a principled approach to constructing outside algorithms, which is a rather straightforward consequence of defining the complement of a substructure relative to the input data object. There are several advantages to this approach:

• One cannot forget contributions to outside recursions. Such missing rules render the algorithm invalid, sometimes in non-obvious ways. This is of particular relevance for complex grammars and when existing algorithms are to be modified.

• Together with the ADPfusion framework the most annoying type of bugs in practical implementations of DP, namely index errors, can be avoided altogether because all index arithmetic is implicit and hidden completely from the user.

• Outside grammar construction is independent of syntactic variable and terminal types. As long as the abstract grammar is a context-free grammar, an outside version can be constructed.

• Our mechanistic construction interacts smoothly with other systems that automate creation of formal grammars, e.g. grammar products [7].

Our current framework still lacks generality and completeness in several respects. It is evident from our example above that data objects of different types can be obtained in the decomposition. For these, parsing may then mean different, type-dependent things. For instance, in the context of forest alignment and forest editing, reviewed in [31], it may be useful to distinguish trees from general forests. This suggests the possibility to develop an algebraic formalism of parsing/decomposition for complex data objects and thus an even higher-level way of specifying the intricacies of parsing schemes underlying DP algorithms. McBride's notion of a derivative operator acting on data types [32] appears to be a relevant starting point in this direction, although it does not seem to be directly applicable.

Although our present framework requires that parsing methods have to be specified for novel data types such as the punctuated sets used in the context of Hamiltonian paths, this has to be done only once and can reused without additional overhead for all DP scenarios on the same data types. In particular, our system already handles all CFGs (and thereby also all linear grammars) on either strings or (punctuated) sets and automatically provides the associated outside algorithms. The high-level framework described here does not require much of a compromise in terms of computational efficiency. While we have to accept a decrease in theoretical performance by a moderate constant factor the gains in ease of algorithm design and actual software development are well worth this price. In the ADPfusion framework we currently have to accomodate approximately a doubling of the running time compared to expert-optimized implementations. Conceptually, the framework extends to multi-contextfree grammars (MCFGs) and thus holds promise to drastically simplify the implementation of algorithms for RNA folding with pseudoknots and complex RNARNA interaction structures. Ongoing work in this area aims at formalizing MCFG-ADP theory [33] and the efficient implementation of the necessary parsers in ADPfusion [34].

## Competing interests

The authors declare that they have no competing interests.

## Authors' contributions

All three authors conceived the study and wrote the manuscript. CHzS implemented the ADP framework, SJP designed and analyzed the gene duplication case study. This work is a revised and extended version of a manuscript presented at the *Brazilian Symposion on Bioinformatics *in Belo Horizonte, Oct 2014 [35].

## Availability

The algorithms described in this work are part of the *generalized ADP *framework. Available here: http://www.bioinf.uni-leipzig.de/Software/gADP/

## References

[B1] Giegerich R Meyer C Kirchner, H., Ringeissen, C Algebraic dynamic programming Algebraic Methodology And Software Technology. Lect. Notes Comp. Sci 2002 2422 Springer, Berlin, Heidelberg 349 364

[B2] Höner zu Siederdissen C Sneaking around concatMap: efficient combinators for dynamic programming Proceedings of the 17th ACM SIGPLAN International Conference on Functional Programming (ICFP'12) 2012 ACM, New York 215 226

[B3] Sauthoff G Janssen S Giegerich R Bellman's GAP - a declarative language for dynamic programming Proceedings of the 13th International ACM SIGPLAN Symposium on Principles and Practices of Declarative Programming (PPDP'11) 2011 ACM, New York 29 40

[B4] Giegerich R Touzet H Modeling dynamic programming problems over sequences and trees with inverse coupled rewrite systems Algorithms 2014 62 144

[B5] Höner zu Siederdissen C Hofacker IL Discriminatory power of RNA family models Bioinformatics 2010 26 453 459 10.1093/bioinformatics/btq370 PMC2935435 20823307

[B6] Voß B Giegerich R Rehmsmeier M Complete probabilistic analysis of RNA shapes BMC Biology 2006 4 5 16480488 10.1186/1741-7007-4-5 PMC1479382

[B7] Höner zu Siederdissen C Hofacker IL Stadler PF Product grammars for alignment and folding IEEE/ACM Trans. Comp. Biol. Bioinf 2014 99 10.1109/TCBB.2014.2326155 26357262

[B8] Rabiner LR A tutorial on hidden markov models and selected applications in speech recognition Proc. IEEE 1989 77 257 286

[B9] Baker JK Trainable grammars for speech recognition J. Acoust. Soc. Am 1979 65 132

[B10] Huang FWD Qin J Reidys CM Stadler PF Partition function and base pairing probabilities for RNA-RNA interaction prediction Bioinformatics 2009 25 2646 2654 19671692 10.1093/bioinformatics/btp481

[B11] Huang FWD Qin J Reidys CM Stadler PF Target prediction and a statistical sampling algorithm for RNA-RNA interaction Bioinformatics 2010 26 175 181 19910305 10.1093/bioinformatics/btp635 PMC2804298

[B12] Reidys CM Huang FWD Andersen JE Penner RC Stadler PF Nebel ME Topology and prediction of RNA pseudoknots Bioinformatics 2011 27 1076 1085 Addendum in: Bioinformatics 28:300 (2012) 21335320 10.1093/bioinformatics/btr090

[B13] Janssen S Kisses, ambivalent models and more: Contributions to the analysis of RNA secondary structure PhD thesis, Univ. Bielefeld 2014 urn: nbn:de:hbz:361-26821318

[B14] Bellman R Dynamic programming treatment of the travelling salesman problem J. ACM 1962 9 61 63

[B15] Held M Karp RM A dynamic programming approach to sequencing problems J. SIAM 1962 10 196 201

[B16] Durbin R Eddy SR Krogh AGM Biological Sequence Analysis 1998 Cambridge University Press, Cambridge

[B17] Needleman SB Wunsch CD A general method applicable to the search for similarities in the amino acid sequence of two proteins J. Mol. Biol 1970 48 443 453 5420325 10.1016/0022-2836(70)90057-4

[B18] Gotoh O Alignment of three biological sequences with an efficient traceback procedure J. theor. Biol 1986 121 327 337 3795999 10.1016/s0022-5193(86)80112-6

[B19] Lorenz R Bernhart SH Höner zu Siederdissen C Tafer H Flamm C Stadler PF Hofacker IL ViennaRNA Package 2.0 Alg. Mol. Biol 2011 6 26 10.1186/1748-7188-6-26 PMC3319429 22115189

[B20] Wuchty S Fontana W Hofacker IL Schuster P Complete suboptimal folding of RNA and the stability of secondary structures Biopolymers 1999 49 2 145 165 10070264 10.1002/(SICI)1097-0282(199902)49:2<145::AID-BIP4>3.0.CO;2-G

[B21] McCaskill JS The equilibrium partition function and base pair binding probabilities for RNA secondary structure Biopolymers 1990 29 1105 1119 1695107 10.1002/bip.360290621

[B22] Dowell RD Eddy SR Evaluation of several lightweight stochastic context-free grammars for RNA secondary structure prediction BMC Bioinformatics 2004 5 71 15180907 10.1186/1471-2105-5-71 PMC442121

[B23] Höner zu Siederdissen C Berhart SH Stadler PF Hofacker IL A folding algorithm for extended RNA secondary structures Bioinformatics 2011 27 129 137 10.1093/bioinformatics/btr220 PMC3117358 21685061

[B24] Höner zu Siederdissen C Hammer S Abfalter I Hofacker IL Flamm C Stadler PF Computational design of RNAs with complex energy landscapes Biopolymers 2013 99 1124 1136 23818234 10.1002/bip.22337

[B25] Elemento O Gascuel O An efficient and accurate distance based algorithm to reconstruct tandem duplication trees Bioinformatics 2002 8 Suppl 2 92 99 10.1093/bioinformatics/18.suppl_2.s92 12385990

[B26] Robinson WS A method for chronologically ordering archaeological deposits Amer. Antiquity 1951 16 293 301

[B27] Prohaska SJ Höner zu Siederdissen C Stadler PF Expansion of gene clusters and the shortest Hamiltonian path problem 2015 10.1007/s00285-017-1197-3 PMC6060901 29260295

[B28] Garcia-Fernàndez J Hox, parahox, protohox: facts and guesses Heredity 2005 94 145 152 15578045 10.1038/sj.hdy.6800621

[B29] Cameron RA Rowen L Nesbitt R Bloom S Rast JP Berney K Arenas-Mena C Martinez P Lucas S Richardson PM Davidson EH Peterson KJ Hood L Unusual gene order and organization of the sea urchin Hox cluster J Exp Zoolog B Mol Dev Evol 2006 306 45 58 10.1002/jez.b.21070 16116652

[B30] Höner zu Siederdissen C Hofacker IL Stadler PF How to multiply Dynamic Programming algorithms Brazilian Symposium on Bioinformatics (BSB 2013). Lect. Notes Bioinf 2013 8213 Springer, Heidelberg 82 93

[B31] Billie P A survey on tree edit distance and related problems Theor. Comp. Sci 2005 337 217 239

[B32] McBride C Clowns to the left of me, jokers to the right (pearl): dissecting data structures 2008 43 287 295

[B33] Riechert M Algebraic dynamic programming for multiple context-free languages Master's thesis, Hochschule für Technik, Wirtschaft und Kultur, Leipzig 2013

[B34] Riechert M Höner zu Siederdissen C Stadler PF Waldmann J Algebraic dynamic programming for multiple context-free languages 2015 in press

[B35] Höner zu Siederdissen C Prohaska SJ Stadler PF Campos, S Dynamic programming for set data types Advances in Bioinformatics and Computational Biology: BSB 2014. Lect. Notes Comp. Sci 2014 8826 57 64

